# Factors Associated with Elevated ALT in an International HIV/HBV Co-Infected Cohort on Long-Term HAART

**DOI:** 10.1371/journal.pone.0026482

**Published:** 2011-11-01

**Authors:** Jennifer Audsley, Eric C. Seaberg, Joe Sasadeusz, Gail V. Matthews, Anchalee Avihingsanon, Kiat Ruxrungtham, Kit Fairley, Robert Finlayson, Hyon S. Hwang, Margaret Littlejohn, Stephen Locarnini, Gregory J. Dore, Chloe L. Thio, Sharon R. Lewin

**Affiliations:** 1 Department of Medicine, Monash University, Melbourne, Australia; 2 Infectious Diseases Unit, Alfred Hospital, Melbourne, Australia; 3 Department of Epidemiology, Johns Hopkins Bloomberg School of Public Health, Johns Hopkins University, Baltimore Maryland, United States of America; 4 National Centre in HIV Epidemiology and Clinical Research, University of New South Wales, Sydney, Australia; 5 HIV-Netherlands-Australia-Thailand Research Collaboration,Bangkok, Thailand; 6 Faculty of Medicine, Chulalongkorn University, Bangkok, Thailand; 7 Melbourne Sexual Health Centre, Melbourne, Australia; 8 Taylor's Square Private Clinic, Sydney, Australia; 9 Victorian Infectious Diseases Reference Laboratory, North Melbourne, Australia; 10 Division of Infectious Diseases, Johns Hopkins University, Baltimore, Maryland, United States of America; 11 Centre for Virology, Burnet Institute, Melbourne, Australia; The University of Hong Kong, Hong Kong

## Abstract

**Background:**

Previous studies have demonstrated that hepatitis B virus (HBV) infection increases the risk for ALT elevations in HIV-HBV co-infected patients during the first year of HAART; however, there is limited data on the prevalence of ALT elevations with prolonged HAART in this patient group.

**Methods/Principal findings:**

To identify factors associated with ALT elevations in an HIV-HBV co-infected cohort receiving prolonged HAART, data from 143 co-infected patients on HAART enrolled in an international HIV-HBV co-infected cohort where ALT measurements were obtained every 6 months was analysed. A person-visit analysis was used to determine frequency of ALT elevation (≥2.5×ULN) at each visit. Factors associated with ALT elevation were determined using multivariate logistic regression with generalized estimating equations to account for correlated data. The median time on HAART at the end of follow-up was 5.6 years (range 0.4–13.3) years. During follow-up, median ALT was 36 U/L with 10.6% of person-visits classified as having ALT elevation. Most ALT elevations were grade 2 (86.5%), with only 13.5% of all ALT elevations grade 3 or higher. Univariate associations with ALT elevation (p<0.05) included history of AIDS, HBV DNA ≥2,000 IU/ml, HBeAg positive, study visit CD4 <200 cells/ml and nadir CD4 <200 cells/ml. In the multivariate analysis, only study visit CD4 <200 cells/ml (OR 2.07, 95%CI 1.04–4.11, *p* = 0.04) and HBeAg positive status (OR 2.22, 95%CI 1.03–4.79, *p* = 0.04) were independently associated with ALT elevation.

**Conclusions:**

In this HIV-HBV co-infected cohort, elevated ALT after >1 year of HAART was uncommon, and severe ALT elevations were rare. HIV-HBV co-infected patients on long-term HAART who are either HBeAg positive or have a CD4 count of <200 cells/ml are at increased risk for ALT elevations.

## Introduction

Approximately 33 million people are infected with human immunodeficiency virus (HIV) [1]. HIV-hepatitis B virus (HBV) co-infection is common due to shared routes of transmission, with reported figures indicating that 6–9% of HIV-infected individuals in developed countries and in Asia are chronically infected with HBV [2]–[4]. HIV infection has a significant impact on the natural history of HBV infection, with increased levels of HBV DNA and an elevated risk of liver-associated mortality [5]–[6].

Co-infection with HBV clearly increases the risk for an elevated alanine aminotransferase (ALT) in patients on HAART [7]. The protease inhibitors are all associated with increased ALT, with high dose ritonavir posing the greatest risk [8]. Non-nucleoside reverse transcriptase inhibitors (NNRTI) have also been linked to hepatotoxicity, particularly nevirapine as part of a hypersensitivity syndrome [9]. The nucleoside reverse transcriptase inhibitors (NRTIs) have the lowest risk but are associated with liver toxicity from steatohepatitis and mitochondrial toxicity [10]. Immune reconstitution has also been recognized as a possible risk factor for elevated ALT following initiation of HAART [11]–[12]. Another contributing factor may be withdrawal of lamivudine (LMV) therapy and/or the development of LMV resistance leading to enhanced replication of HBV [13]–[14]. Finally, immune escape mutants of HBV, including mutations that reduce synthesis of HBeAg or pre-core mutants, may be selected causing progressive liver damage [12]. 

With prolonged exposure to HAART, HIV-HBV co-infected patients may have an increasing risk of ALT elevations due to longer duration of hepatotoxic drugs and an increased immune response to HBV antigens. Alternatively, the risk may decrease with longer HAART especially if HBV-active drugs effectively control HBV replication. In order to determine the factors associated with elevated ALT among HIV-HBV co-infected patients on HAART longer than one year, we studied a prospectively-followed international cohort of HIV-HBV co-infected patients.

## Materials and Methods

### Ethics statement

Written, informed consent was obtained from all participants, and the study was approved by the relevant Human Research Ethics Committees in Australia, the United States and Thailand. This study was conducted according to the principles expressed in the Declaration of Helsinki.

### Study participants

169 HIV/HBV co-infected individuals were enrolled from sites in Australia (The Alfred Hospital, The Royal Melbourne Hospital and Melbourne Sexual Health Clinic, Melbourne; St Vincent's Hospital and Taylor's Square Clinic, Sydney); the United States (The Multicenter AIDS Cohort Study - MACS) and in Thailand (HIV-NAT, Thai Red Cross AIDS Research Centre, Bangkok) over the period October 2004 to February 2008. Eligibility criteria have been previously reported [15]. Individuals with chronic hepatitis C virus (HCV; HCV antibody and HCV RNA positive at study entry) were not eligible. For inclusion in this analysis, we selected only those time points at which a patient was on HAART, had a positive HBsAg and had ALT assessed. Thus, this study included 701 person-visits from 143 patients.

### Data abstraction and collection

Clinical and laboratory data were collected or abstracted from medical records at study entry and at 6-monthly follow-up visits. Clinical data included demographics, prior and current anti-HIV and anti-HBV therapy, previous/present AIDS-defining illnesses, history/current jaundice, hepatocellular carcinoma (HCC), ascites, oesophageal varices, hepatic encephalopathy and Child-Pugh stage. Laboratory measurements included ALT, aspartate aminotransferase (AST), international normalized ratio (INR) or prothrombin time, haemoglobin, white blood cell count, platelets, hepatitis B e antigen (HBeAg), HBe antibody (anti-HBe), HB surface antibody (anti-HBs), HIV RNA, CD4 count at the time of study visit, and nadir CD4 count. Hepatitis delta virus (HDV) was collected at study entry only. Data on alcohol intake and compliance to HAART were also collected at each visit. Patients in the US and Australia accrued up to four years of follow-up and those from Thailand up to 18 months.

### Laboratory testing

HBV DNA was quantified using the RealART™ HBV LC PCR (QIAGEN), lower limit of detection (LLOD) 20 IU/ml, in accordance with the manufacturer's instructions.

HIV RNA was quantified by the approved, standard test used at each site, which were performed according to manufacturer's instructions. For analysis, HIV RNA was classified as detectable (≥400 copies/ml) or undetectable (<400 copies/ml).

### Statistical analysis

This longitudinal study included data from up to 9 visits for each participant. At each study visit elevated ALT was defined using the ACTG criteria: grade 0 (<1.25×upper limit of normal, ULN); grade 1 (1.25–2.5×ULN); grade 2 (>2.5–5.0×ULN); grade 3 (>5.0–10.0×ULN); and grade 4 (>10.0×ULN)) where ALT ULN was set at 30 U/L for men and 19 U/L for women based on Prati et al [16]. Clinical and laboratory variables examined in the analyses included study visit, recruitment site, gender, age >40 years, injecting drug use (IDU) at study entry, men who have sex with men (MSM) contact, heterosexual contact, previous AIDS-defining illness, HBV DNA ≥2000 IU/ml, HBeAg status, anti-HBe status, HBV-active antiretrovirals (ARVs), duration on HAART, current ARVs, detectable HIV RNA (>400 copies/ml), current and nadir CD4 cell count, median weekly alcohol intake and the components of the Child-Pugh score. The HBV DNA cut-off used in this analysis was ≥2,000 IU/ml since it had previously been shown to be associated with ALT elevation in the first 18 months of HAART [17].

Standard descriptive statistics (e.g., frequency, percentages) were used to characterize the study cohort at study entry. The prevalence and cumulative incidence of grade 2 or higher ALT elevation and the change in median ALT over time were displayed graphically. Multiple logistic regression with robust variance estimation was used to determine characteristics associated with ALT elevations of grade 2 or higher while accounting for within-subject correlation [18]. To account for the prospective study design and the fundamental differences between the cohorts, we forced covariates for study visit, study site and gender into all multivariate models. Observations with missing data were included in the multiple regression analyses using multiple imputation [19]. All statistical analyses were performed using SAS 9.2 (SAS Institute, Cary, NC), and statistical significance was defined as a p-value <0.05.

## Results

### Baseline characteristics of study subjects

The majority of the cohort was male (90.2%), aged over 40 years (62.9%) and with a HIV risk factor of MSM (71.3%), as summarized in Table 1. HBV DNA was detected in 21.0% and HIV RNA was detected in 15.8% of patients. About half the cohort was HBeAg positive (50.8%). The median time on HAART since HAART initiation at enrolment to this study and at the end of follow-up was 3.5 yrs (range: 0.3–10.8 yrs) and 5.6 (0.4 to 13.3) years respectively. Sixty-two percent of the patients were receiving a non-nucleoside reverse transcriptase inhibitor and 38% a protease inhibitor. Ninety-three per cent of those on HAART had HBV-active agents as part of their HAART regimen, which included lamivudine (LMV) or emtricitabine (FTC; 18.2%), tenofovir disoproxil fumarate (TDF; 13.3%) and TDF with LMV or FTC (61.5%). Baseline CD4 was <200 cells/µl in a minority of cases (16.5%) with a median CD4 count of 389 cells/µl. The majority of the cohort had a mean alcohol intake of <14 standard drinks/week (92.8%) and a Childs-Pugh score of 5 (88.9%).

**Table 1:** Study entry demographics and clinical characteristics.

Characteristic	Number (%)
**Participants by location** (n = 143)	
Australia	61 (42.7)
MACS	39 (27.3)
Thailand	43 (30.0)
**Gender**, m^1^/f	129 (90.2)/14 (9.8)
**Age >40 years**	90 (62.9)
2 **HIV risk factor**	
Ever IDU	15 (10.7)
Heterosexual contact	32 (22.4)
MSM contact	102 (71.3)
**History of AIDS**	53 (37.1)
**HBeAg status**, positive/negative (n = 134)	68 (50.8)/66 (49.2)
**Anti-HBe status**, positive/negative (n = 103)	42 (40.8)/61 (59.2)
**Detectable HBV DNA** (≥2,000 IU/ml) (n = 138)	29 (21.0)
**Detectable HIV RNA** (≥400 copies/ml) (n = 139)	22 (15.8)
**Study entry CD4 <200 cells/ml** (n = 138)	23 (16.5)
**Nadir CD4 <200 cells/ml** (n = 141)	88 (62.4)
**Child-Pugh score** (n = 117)	
5	104 (88.9)
6	10 (8.6)
7–9	3 (2.6)
**Alcohol intake (mean drinks/week)** (n = 140)	
none	45 (32.1)
<14	85 (60.7)
≥14	10 (7.1)
**Current ARVs**	
NRTI	137 (95.8)
PI	54 (37.8)
NNRTI	89 (62.2)
HBV-active ARVs	133 (93.0)
**HBV active HAART**	
None	10 (7.0)
LMV/FTC only	26 (18.2)
TDF only	19 (13.3)
TDF+LMV/FTC	88 (61.5)

1 for analysis, male category includes 1 transgender M->F individual.

2 some participants report multiple risk factors.

MACS: Multicenter AIDS Cohort Study; IDU: intravenous drug use; ARV: antiretroviral agent; HAART: highly active antiretroviral therapy; MSM: men who have sex with men; HBeAg/Ab: hepatitis B e antigen/antibody; NRTI: nucleoside reverse transcriptase inhibitor; PI: protease inhibitor; NNRTI: non-nucleoside reverse transcriptase inhibitor; LMV: lamivudine; FTC: emtricitabine; TDF: tenofovir disoproxil fumurate.

### Elevated ALT

**Figure 1:**
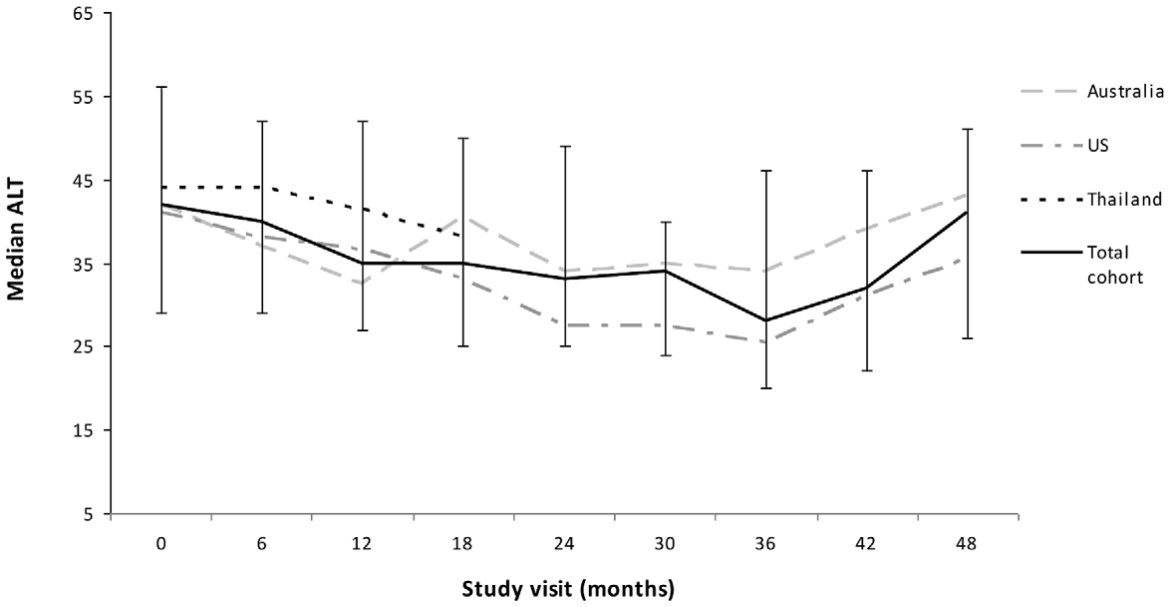
Median ALT data summary by person-visits over the study duration.

**Figure 2:**
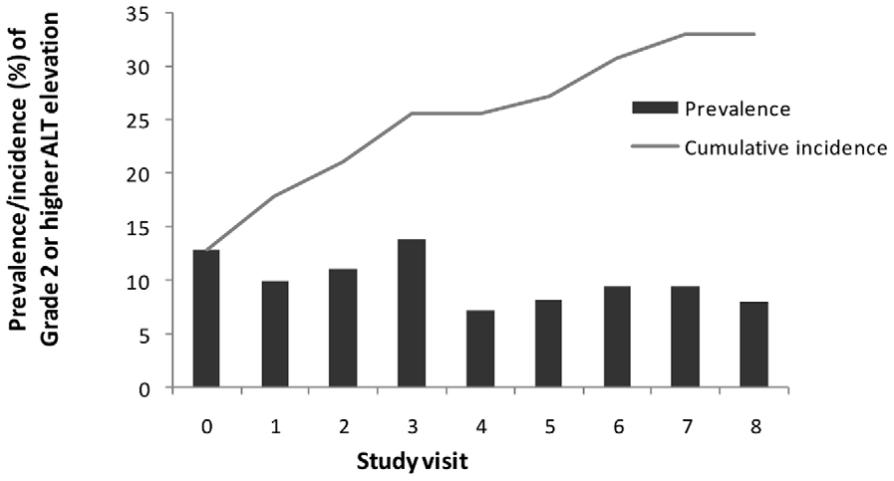
Prevalence and cumulative incidence of grade 2 or higher ALT elevation during the study period.

Across the 701 person-visits, the median ALT was 36 (IQR 26–50) and notably, it remained relatively stable throughout follow-up (Figure 1). Only 74 person-visits (10.6%) were classified as having grade 2 or higher ALT elevation of which the majority were grade 2 (86.5%, n = 64) and grade 3 and 4 elevations occurred in 10.8% (n = 8) and 2.7% (n = 2) of visits, respectively. The probability of a grade 2 or higher ALT elevation remained stable about 10% throughout follow-up (Figure 2). The cumulative incidence reached 33% by the end of the study period (Figure 2). In contrast the cumulative incidence of grade 3 or higher elevation was low at 8.7% (data not shown).

The majority of the ALT elevations (78.4%) were in patients who had more than one visit with grade 2 or higher ALT elevation. Three of these patients had elevated ALT for at least 2 years (5 consecutive study visits). We were unable to determine if these persistent elevations of ALT were associated with adverse outcomes.

There was no association between elevated ALT and anti-HBe seroconversion. Anti-HBe seroconversion was observed in a total of 10 patients but elevated ALT was observed prior to anti-HBe seroconversion in only one patient. In this patient, ALT elevation continued following seroconversion. In the other 9 patients, there was no change in ALT either prior to or following anti HBe seroconversion

Univariate analysis (Table 2) identified that a history of AIDS-related illness, HBV DNA >2,000 IU/ml, HBeAg positive status, CD4 count <200 cells/ml, and a nadir CD4 count <200 cells/ml were significantly associated with ALT elevation. Notably, HAART duration longer than 1 year was not associated with risk of ALT elevation. In the multivariate analysis only CD4 count <200 cells/ml (OR 2.07, *p* = 0.04) and HBeAg positive status (OR 2.22, *p* = 0.04) remained independently associated with increased risk for elevated ALT (Table 3).

**Table 2:** Associations of selected cohort characteristics with elevated ALT^1^.

Variable	All follow-up					
		Person-visit, n (%)	Median ALT	IQR	%elevated ALT^1^	*p*
**History of AIDS**	Yes	252 (36.0)	43	27–61	17.1%	**0.04**
	No	599 (85.4)	34	25–46	6.9	
**HBV DNA**	≥2,000 IU	92 (15.1)	44	34–64	17.4	**0.01**
	<2,000 IU	519 (84.9)	35	25–49	9.4	
**HBeAg**	Positive	299 (45.3)	41	30–58	15.1	**0.001**
	Negative	361 (54.7)	33	24–45	7.5	
**HBV-active ARVs**	None	14 (2.0)	44	30–84	28.6	0.3
	LMV/FTC only	102 (14.6)	40	27–52	13.7	
	TDF only	61 (8.7)	31	25–46	4.9	
	TDF+LMV/FTC	524 (74.8)	35	25–50	10.7	
**Current NRTI**	Yes	692 (98.7)	36	26–60	10.3	0.14
	No	9 (1.3)	43	34–90	33.3	
**Current PI**	Yes	340 (48.5)	35	25–49	12.9	0.20
	No	361 (51.5)	38	27–50	8.3	
**Current NNRTI**	Yes	390 (55.6)	38	22–49	8.5	0.09
	No	311 (44.4)	35	25–52	13.2	
**Alcohol intake** 2	None	212 (30.5)	38	27–51	9.4	0.75
	<14 std drinks	442 (63.6)	35	25–49	10.2	
	≥14 std drinks	41 (5.9)	43	25–66	22.0	
**CD4 - study visit**	<200 cells/ml	100 (14.5)	42	27–66	20.0	**0.002**
	≥200 cells/ml	591 (85.5)	35	25–49	9.1	**0.03**
**Nadir CD4**	<200 cells/ml	412 (60.0)	38	26–55	13.8	
	≥200 cells/ml	275 (40.0)	34	25–44	4.4	
**Child-Pugh score**	5	506 (87.1)	37	26–49	9.9	0.15
	6	46 (7.9)	43	29–77	28.3	
	7–9	29 (5.0)	37	27–46	10.3	

1 Grade 2 or higher (ACTG criteria), ULN of ALT was 30 for men and 19 for women.

2 median weekly standard drinks.

IDU: intravenous drug use; ARV: antiretroviral; HAART: highly active antiretroviral therapy; HBeAg/Ab: hepatitis B e antigen/antibody; LMV: lamivudine; TDF: tenofovir disoproxil fumarate; FTC: emtricitabine.

Additional variables analysed at the univariate level that were not statistically significant included: recruitment site location, gender, study visit, ever IDU, MSM contact, heterosexual contact, anti-HBe status, and detectable HIV RNA.

**Table 3:** Multiple regression analysis – logistic regression (outcome elevated ALT).

	Multivariate Model		
Co-variate	OR	95% CI	*p*
Study visit	0.95	0.86–1.05	0.35
MACS vs. Thai site	1.29	0.33–5.0	0.72
Australia vs. Thai site	2.03	0.60–6.9	0.26
Female	2.00	0.40–10.1	0.40
HBe Ag positive	2.22	1.03–4.79	**0.04**
HBV DNA ≥2,000 IU/ml	2.02	0.84–4.83	0.11
CD4 <200 cells/ml at time of study visit	2.07	1.04–4.11	**0.04**

HBeAg: hepatitis B e antigen.

## Discussion

This study is the first designed to specifically examine ALT elevations in a large, long-term cohort of HIV-HBV co-infected patients on >1 year of HAART. We found that in patients on HAART for a median time of over 5years, ALT remained relatively stable, the probability of ALT elevations was low and did not increase over time and most ALT elevations were accounted for by patients with more than one episode of ALT elevation. The estimated cumulative incidence of grade 2 or higher ALT elevations was 33% over 4 years and for grade 3 or higher was only 8.7%. Patients with more advanced HIV (follow-up visit CD4 count <200cells/ml) and those who were HBeAg positive were at the greatest risk for ALT elevations on long-term HAART.

Elevated ALT is generally regarded as a marker of hepatic necrosis and inflammation, although liver damage may be present with normal ALT [5], [20]. It is encouraging that ALT elevations in HIV-HBV co-infected patients on HAART over several years was low and did not increase with duration of HAART. Only three patients had elevated ALT for a prolonged period (at least 2 years). Several observational cohort studies have shown that the incidence of ALT elevations on HAART ranged from 5–45%, and varied based on the definition used including any elevation above normal, mild elevation (up to and including Grade 2 i.e. >2.5–5×ULN) and severe elevation (Grade 3 >5<10× and/or Grade 4 >10×ULN). In addition, most published observational cohort studies of patients with hepatitis co-infection have included both HCV and HBV co-infection with a higher percentage of patients co-infected with HCV than HBV [7]–[8], [17], [21]–[31]. Follow up in these studies ranged from 6 months to 4.8 years (median 17.8 months) and common factors associated with ALT elevations included ritonavir or nevirapine-containing regimens and baseline ALT [8], [25]–[27], [31]–[32]. These studies concentrated on either treatment-naïve patients commencing HAART or PI-naïve patients commencing PI-containing regimens, and suggested that the highest risk of elevated ALT was early following initiation of HAART. In contrast, in our study 94.4% of patients had been receiving HAART for longer than 12 months. One previously published observational cohort describing liver disease included only HIV-HBV co-infected patients and reported that patients with mean transaminases above the ULN were significantly more likely to develop advanced liver disease [29].

A significant association between lower CD4 count and elevated ALT has not been consistently reported in previous studies. In one study of patients with HIV-HBV co-infection there was no significant association between mean CD4 count during follow up and the development of advanced liver disease [29], while Sulkowski *et al*. reported that a larger CD4 cell count increase was associated with severe hepatotoxicity (ALT >5×ULN) in HIV-hepatitis co-infection [7]. In studies of HIV mono-infected patients that exclude viral hepatitis, one study has reported an increased risk of ALT with lower CD4 while another study reported the opposite findings [33]–[34]. We were surprised to find this association in the setting of HIV-HBV co-infection given our previous work showing that in HIV-HBV co-infected patients not receiving HAART, a lower CD4 count was associated with a lower number of HBV-specific T-cells [35] and that there was little increase in HBV-specific T-cells in the first 48 weeks following HBV-active HAART [11]. However, elevated ALT can occur secondary to both the adaptive and innate immune responses (summarised in [36]). It is possible that other factors might be driving an increase in ALT, even in patients with well controlled HBV DNA, such as circulating lipopolysaccharide (LPS) in HIV-infected patients which is higher in patients with low CD4 T-cell counts [37]–[38]. Elevated LPS can increase Kupffer cell activation [39], leading to liver disease progression as described in HCV infection, HIV-HCV co-infection and alcoholic liver disease [40]–[42] and we have recently demonstrated that LPS is also significantly elevated in HIV-HBV co-infection [43].

HBeAg positive status represents greater HBV replication and is considered a surrogate marker of elevated HBV DNA in untreated patients. HBV DNA is usually higher in HBeAg positive than in HBeAg negative disease [44]. Rates of HBeAg loss and/or seroconversion are low following HBV-active NRTI, even in the setting of prolonged suppression of HBV DNA [45]–[46]. In this study, both HBeAg positive status and HBV DNA ≥2,000 IU/ml were significantly associated with elevated ALT at the univariate level, however only HBeAg status remained significant when both HBeAg and HBV DNA were included in the multivariate model. These findings may be explained by the low number of patients with HBV DNA above 2,000 IU/ml (15%) compared with almost half the cohort (45%) being HBeAg positive over the follow-up period. HBeAg could also be a surrogate for duration of infection with HBV, a variable we were unable to collect in this cohort. In general, patients who are HBeAg positive are infected for a shorter duration of time than those who are HBeAg negative [47] and are therefore more likely to enter an immunoactive phase which may contribute to an elevated ALT.

There are several limitations to this study. First, it is possible we may have underestimated the prevalence of an elevated ALT in our cohort as patients only had ALT collected every 6 months. There were 182 person-visits excluded from the analysis due to missing ALT data. However, we think this was unlikely to have had an effect on the final results because of the large overall sample size. Second, our use of the revised more stringent guidelines for defining normal ALT may have increased the prevalence of an elevated ALT; however, we considered that this definition was appropriate given that previous studies showed ALT levels are lower in co-infection even in the setting of liver disease [6] and that they are the currently accepted normal levels of ALT in patients with HBV mono-infection [AASLD guidelines [48]]. Third, we did not measure HCV RNA or HDV Ab or RNA throughout follow up. Given the high frequency of MSM in this cohort, acute HCV could be a possible explanation of elevated ALT, although we have no evidence for this. In contrast, there is a low prevalence of HDV infection in Australia and Thailand ([49]–[50]; personal communication, Scott Bowden, Victorian Infectious Diseases Reference Laboratory) so we think this would be an unlikely cause of elevated ALT. Fourth, we recruited patients from three geographically different sites; therefore the HBV genotype distribution and ethnicity were different; however, recruitment site was not a significant factor in any of the analyses. Finally, we did not directly assess liver disease severity, other than the Childs-Pugh scores. The Childs-Pugh scores for the cohort ranged from 5–7 throughout follow-up, which is indicative of relatively mild liver disease in the cohort, and therefore our results might not be applicable to cohorts with more advanced liver disease.

In conclusion, in a large prospective cohort of HIV-HBV co-infected patients on HAART for up to 13.3 years at the end of follow up, the prevalence of elevated ALT was low and was stable across study visits. CD4 count <200 cells/ml and HBeAg positive status were significantly associated with an increased risk of elevated ALT; thus HIV-HBV co-infected patients with these characteristics require careful monitoring of ALT.
